# Strangulated Littre's direct inguinal hernia: a case report and literature review

**DOI:** 10.1093/jscr/rjaf416

**Published:** 2025-07-03

**Authors:** Ahmed Khamas, Salah Salih

**Affiliations:** Department of Continuing Medical Education, Garmian Health Directorate, Kalar, Sulaymaniyah 46021, Iraq; Department of General Surgery, Baquba Teaching Hospital, Baquba, Diyala 32001, Iraq

**Keywords:** Littre’s hernia, Meckel’s diverticulum, inguinal hernia, complication, strangulation, incarceration

## Abstract

The rare occurrence of a Meckel's diverticulum within a hernial sac, known as a Littre’s hernia, was first described by the French anatomist and surgeon Alexis Littré in the 18^th^ century. This condition is observed in only 0.09% of strangulated hernias. Patients typically present with an irreducible lump in the groin. In this case report, we present a 66-year-old man who sought medical attention with a 72-hour history of an incarcerated inguinal hernia. Patient underwent hernia repair and intraoperatively, strangulated Meckel's diverticulum, adhered medially to the base of the sac, was identified. The patient was discharged after an uneventful recovery. This case underlines the importance for surgeons to consider Littre's hernia as a potential differential diagnosis, even in patients over 60 years old who present without overt gastrointestinal symptoms. Maintaining a high index of suspicion for this rare type of hernia can help guide appropriate diagnostic evaluation and management.

## Introduction

The rare occurrence of a Meckel's diverticulum within a hernial sac, first described by the French anatomist and surgeon Alexis Littré in the 18th century, is known as Littre’s hernia [1]. It is a rare surgical condition with < 60 cases documented in the literature [2].

Littre’s hernias, an unusual complication of Meckel's diverticulum, account for 0.09% of all strangulated groin hernias and commonly occur in right inguinal region [3–8].

Meckel’s diverticulum, the most common deformity of the gastrointestinal tract, can be found in ~2% of the population [1, 7]. Complications of Meckel’s diverticulum (4%–6%) commonly occurred in children <2 years old [6, 7]. We are reporting a case of a right-sided incarcerated and strangulated direct inguinal Littre’s hernia in a man over his mid-60s.

## Case report

In July 2013, a 66-year-old Kurdish male presented to the emergency department at general hospital with right-sided painful inguinal swelling for the last 3 days. The swelling and pain gradually increased and accompanied by vomiting for one time and anorexia suspicious of complicated right inguinal hernia. Initially, pain was colicky in nature then became constant. The pain radiated to the peri-umbilical area and was worse on standing and relieved by lying supine. Furthermore, the patient had no history of recurrent abdominal pain or rectal bleeding.

The patient general physical examination was unremarkable except for regular tachycardia of 110 beats per minutes. On local examination, a tender irreducible lump at the right inguinal region was identified. Laboratory investigations showed leucocytosis of 12 000 cells per mm^3^. Ultrasound showed a hernial sac with bowel loop at the right inguinal canal with fluid in the peri sac region. Diagnosis of strangulated right-sided direct inguinal hernia was made. The patient admitted and has been properly prepared for the open surgery.

**Figure 1:**
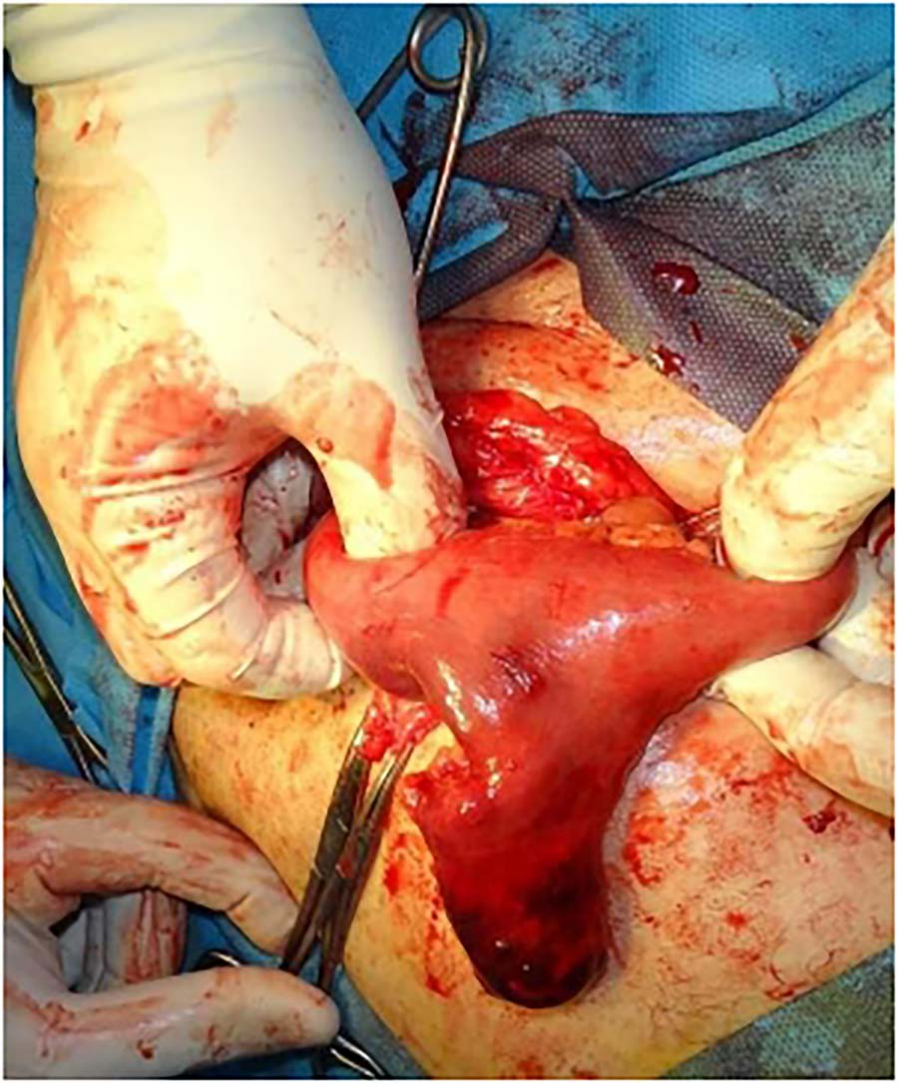
Intraoperative photograph after opening the hernial sac, showing the incarcerated Meckel's diverticulum.

**Figure 2:**
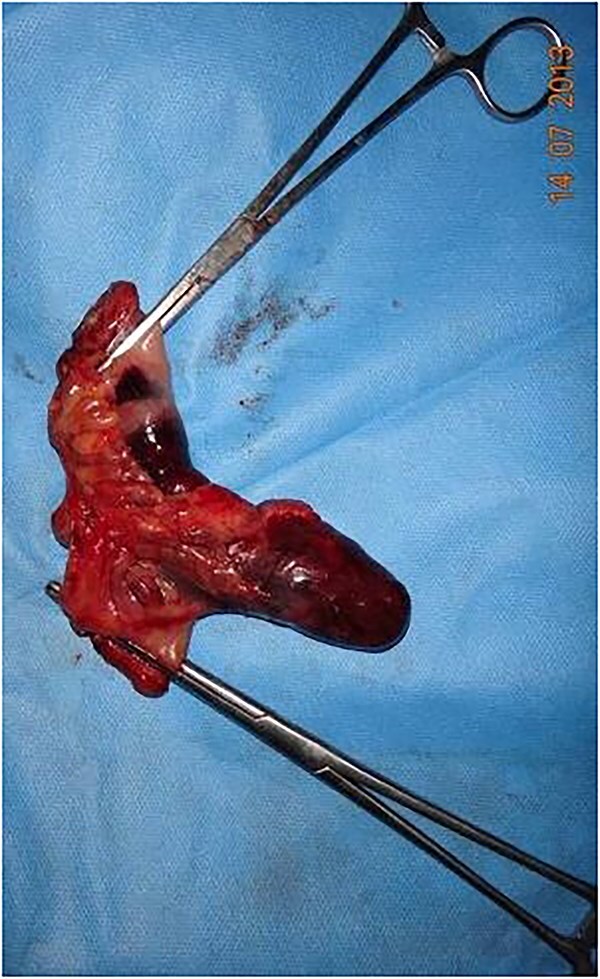
Surgically resected Meckel's diverticulum shown.

Intraoperatively, an incarcerated and strangulated Meckel's diverticulum, adhered medially to the base of the sac, was identified (Fig. 1) and resection with an end-to-end anastomosis was done (Fig. 2). Posterior wall of the inguinal canal was repaired by Nylon-darn herniorrhaphy.

After an uneventful recovery, patient was discharged on the 4th postoperative day and sutures were removed on 7th postoperative day. The histopathological examination revealed Meckel's diverticulum and there was no ectopic tissue. The patient remains asymptomatic 1 year after the operation.

## Discussion

Littre’s hernia, a rare perioperative entity, is the projection of a Meckel's diverticulum through a potential abdominal opening [1]. A Meckel's diverticulum can be the sole contents of the hernia sac, or it may be accompanied by the adjacent loop of the ileum [1]. Meckel's diverticulum, a common gastrointestinal tract deformity, remains totally asymptomatic, or it can be discovered incidentally in 1%–2% of the population with equal sex ratio, however this is not the case in symptomatic Meckel's as tendency of having complication is higher in male [6]. The Incidence of Littre’s hernias is more on the right side, taking in consideration that inguinal hernias are more common in males and that Meckel diverticula have been reported to occur in inguinal region in 50% of cases [1, 5, 7, 8].

The diverticulum can adhere to the wall of a hernial sac this occurs when more bowels enter the sac, causing the apex of the diverticulum to point towards the sac’s neck. This results in kinking and twisting of the diverticulum's base, leading to obstruction. Consequently, the entrapped diverticulum may become incarcerated or even strangulated, though this is a relatively uncommon occurrence [1].

Littre’s hernia usually presents as a lump in the groin with almost entire absence of mechanical obstruction [1]. The Mayo clinic experience with 1467 patients who had had Meckel's diverticulum from 1950 to 2002 showed that 16% had a symptomatic Meckel’s, of them only two cases were presented with a hernia of incarcerated Meckel's [9]. In addition, male sex and age <50 were found to be frequent in these symptomatic cases [9].

Only one case of an indirect strangulated right inguinal Littre’s hernia was previously documented for a male patient over 60-year-old [7, 10]. In the present case, our patient was the first case for a 66-year-old male in Iraq.

With Litter’s hernia preoperative diagnosis rendered unachievable [3–5], all previous case reports described how difficult it is for both surgeons and radiologists to have accurate diagnose before surgery [1–12], and this was the case in our patient as well. When identified during surgery, Meckel's diverticulum resection should be performed if the mouth base is not wide and if its wall is thickened [5, 12], incarceration was the reason for resection and end to end anastomosis in this case.

In conclusion, this case highlighted that it is important for surgeons to think about Littre’s hernia as a differential diagnosis in male with age even higher than 60 years-old even with no gastrointestinal manifestations.
